# The Inhibition of HIV-1 Entry Imposed by Interferon Inducible Transmembrane Proteins Is Independent of Co-Receptor Usage

**DOI:** 10.3390/v10080413

**Published:** 2018-08-07

**Authors:** Jingyou Yu, Shan-Lu Liu

**Affiliations:** 1 Center for Retrovirus Research, The Ohio State University, Columbus, OH 43210, USA; yu.2123@osu.edu; 2 Department of Veterinary Biosciences, Department of Microbial Infection and Immunity, The Ohio State University, Columbus, OH 43210, USA; 3 Viruses and Emerging Pathogens Program, Infectious Diseases Institute, The Ohio State University, Columbus, OH 43210, USA

**Keywords:** IFITM, HIV-1, entry, co-receptor

## Abstract

Interferon inducible transmembrane proteins (IFITMs) are one of several IFN-stimulated genes (ISGs) that restrict entry of enveloped viruses, including flaviviruses, filoviruses and retroviruses. It has been recently reported that in U87 glioblastoma cells IFITM proteins inhibit HIV-1 entry in a co-receptor-dependent manner, that is, IFITM1 is more inhibitory on CCR5 tropic HIV-1 whereas IFITM2/3 confers a greater suppression of CXCR4 counterparts. However, how entry of HIV-1 with distinct co-receptor usage is modulated by different IFITM orthologs in physiologically relevant CD4^+^ T cells and monocytes/macrophages has not been investigated in detail. Here, we report that overexpression of IFITM1, 2 and 3 in human CD4^+^ HuT78 cells, SupT1 cells, monocytic THP-1 cells and U87 cells expressing CD4 and co-receptor CCR5 or CXCR4, suppressed entry of CXCR4 tropic viruses NL4.3 and HXB2, CCR5 tropic viruses AD8 and JRFL, dual tropic 89.6 virus, as well as a panel of 32 transmitted founder (T/F) viruses, with a consistent order of potency, that is, IFITM3 > IFITM2 > IFITM1. Consistent with previous reports, we found that some CCR5-using HIV-1 isolates, such as AD8 and JRFL, were relatively resistant to inhibition by IFITM2 and IFITM3, although the effect can be cell-type dependent. However, in no case have we observed that IFITM1 had a stronger inhibition on entry of any HIV-1 strains tested, including those of CCR5-using T/Fs. We knocked down the endogenous IFITMs in peripheral blood mononuclear cells (PBMCs) and purified CD4^+^ T cells and observed that, while this treatment did greatly enhance the multiple-round of HIV-1 replication but had modest effect to rescue the single-round HIV-1 infection, reinforcing our previous conclusion that the predominant effect of IFITMs on HIV-1 infection is in viral producer cells, rather than in target cells to block viral entry. Overall, our results argue against the idea that IFITM proteins distinguish co-receptors CCR5 and CXCR4 to inhibit entry but emphasize that the predominant role of IFITMs on HIV-1 is in producer cells that intrinsically impair the viral infectivity.

## 1. Introduction

Interferon inducible transmembrane proteins (IFITMs) have been shown to be essential restriction factors against a broad spectrum of enveloped viruses (reviewed in [1,2]), including human immunodeficiency virus type 1 (HIV-1) [3]. Recently, we and others discovered that IFITMs not only block HIV-1 entry into target cells but also and more potently, diminish HIV-1 infectivity in viral producer cells [4,5,6]. The current proposed model for the mechanism of action of IFITM proteins on HIV-1 infectivity includes negative imprinting, in which IFITMs are incorporated into virions [4,5], as well as impaired HIV-1 envelope (Env) processing [6]. Subsequent investigations showed that IFITM proteins can distinctly inhibit HIV-1 with different tropisms; in particular, it was shown that some CCR5 tropic viruses, including those of transmitted founders (T/Fs), were relatively resistant to IFITM-mediated impairment of HIV-1 infectivity and entry [7,8]. Interestingly, Neil and colleagues reported that IFITM1 is more potent to restrict entry of CCR5 viruses whereas IFITM2 and IFITM3 are more effective to block entry of CXCR4 viruses, at least in U87 cells [8]. These results are apparently different from some of the earlier reports in Jurkat cells, SupT1 cells and HeLa TZM-bl cells [6,9], where IFITM2 and IFITM3 were shown to be generally more potent than IFITM1 to inhibit HIV-1, regardless of co-receptor usage.

To further determine the roles of IFITMs in HIV-1 entry into target cells, as well as to resolve some of the discrepancies, herein we have performed comprehensive analysis of a series of HIV-1 strains, including two CXCR4-tropic strains NL4.3 and HXB2, two CCR5-tropic viruses AD8 and JRFL, one dual tropic 89.6, as well as twenty HIV-1 B-clade Env reference clones and twelve global T/F viruses. To interrogate the role of endogenous IFITMs, we knocked down IFITMs in human PBMCs and purified CD4^+^ T cells and determined the contribution of IFITMs in IFN-mediated inhibition of multiple and single round of HIV-1 infection. Our results showed that IFITM proteins do not distinguish co-receptors CCR5 and CXCR4 to inhibit HIV-1 entry but highlight a more important role of IFITMs in diminishing viral infectivity.

## 2. Materials and Methods

### 2.1. Cell Lines and Reagents

U87.CD4.CXCR4 (Cat# 4036) and U87.CD4.CCR5 (Cat# 4035) cells were obtained from the NIH AIDS Reagent Program. Drs. Li Wu (The Ohio State University, Columbus, OH, USA) and Eric Freed (NCI, Frederick, MD, USA) kindly provided HuT78/CCR5 cells and peripheral blood mononuclear cells (PBMCs), respectively. U87 and HEK293T cells were maintained in DMEM (Hyclone) supplemented with 10% fetal bovine serum (FBS) and 100 U/mL penicillin and streptomycin, while HuT78/CCR5 and PBMCs were maintained in RPMI1640 (Hyclone) supplemented with 10% FBS and 100 U/mL penicillin and streptomycin.

Human CD4^+^ T lymphocytes were isolated from PBMCs by negative autoMACS selection using a CD4^+^ T lymphocytes isolation kit (Miltenyi Biotec, Bergisch Gladbach, Germany). The purity of CD4^+^ T lymphocytes was above 90% after autoMACS separation as validated by CD4 staining using flow cytometry. CD4^+^ T cells were activated with PHA-P (2 µg/mL) for 2 days and then maintained in RPMI1640 with 20 U/mL interleukin-2 till use.

Antibodies against IFITM1 (Cat# 11727-3-AP), IFITM2 (Cat# 12769-1-AP) and IFITM3 (11714-1-AP) were purchased from Proteintech Group (Chicago, IL, USA), while anti-FLAG M2 antibody was purchased from Sigma (St. Louis, MO, USA).

*Plasmids.* pQCXIP/pQCXIH empty vector and FLAG-IFITM1, 2, 3 or untagged wildtype (WT) expression plasmids have been reported previously [10]. ShRNA control and shRNA IFITM1, 2, 3 constructs were purchased from Sigma. The panel of SGA HIV-1 Subtype B T/F Env Clones (Cat# 11663, contributed by Drs. Beatrice H. Hahn, Brandon F. Keele and George M. Shaw) and the panel of global HIV-1 Env clones (Cat# 12670, contributed by multiple international investigators [11]) were obtained from the NIH AIDS Reagent Program. Amphotropic MLV 10A1 Env has been previously described [10,12] and NL4.3 Env and AD8 Env constructs were kindly provided by Dr. Eric Freed. 89.6 Env was kindly provided by Dr. Jesse Kwiek (The Ohio State University, Columbus, OH, USA). HXB2 and JRFL Env were obtained from Dr. Chen Liang’s lab (McGill University, Montreal, QC, Canada).

### 2.2. Virus Production

One-round HIV-1 GFP reporter viruses were produced by co-transfection of pLenti-puro-GFP, HIV-1 gag-pol Δ8.2 (containing all accessory and regulatory genes) and HIV-1 Env plasmids at ratio of 1:1:0.5 into HEK293T cells. Similarly, intron gaussia luciferase reporter viral stocks were made by transfecting pHIV-inGLuc, HIV-1 Gag-pol Δ8.2 and HIV Env at ratio of 1:1:0.5 into HEK293T cells. Lentiviral shRNA viral stocks (by using pLenti-shRNA, HIV gag-pol Δ8.2 and pMDG) or pQCXIP/pQCXIH (by using pQCXIP, MLV gag-pol and pMDG)-based retroviral stocks were similarly produced at ratio of 1:1:0.5 into HEK293T cells. Twenty-four hours after transfection, supernatants were collected and filtered through 0.22 µm filter and stored at −80 °C until use.

### 2.3. Stable Cell Line Generation

For suspension cells, 5 × 10^5^ HuT78/CCR5, SupT1, or THP-1 cells were seeded onto 6-well plates; the next day, 400 µL viral stocks were applied to each well together with 5 µg/mL polybrene by spinoculation at 1680 g, 4 °C for 1 h. For U87-based cells, 1 × 10^5^ cells were seeded onto 6-well plates overnight and 200 µL viruses were added into each well mixed with 5 µg/mL polybrene; 24 h after transduction, DMEM or RPMI1640 medium containing 1 µg/mL puromycin or 250 µg/mL hygromycin was applied for selection up to 2 weeks until the negative control cells were completely dead.

### 2.4. Virus Infection

For adherent U87 cells, GFP or luciferase reporter viruses were directly applied to 12-well plates. For suspension cells, reporter viruses were applied to each well and spinoculated for 1 h at 1680 g, 4 °C. Six hours after transduction, cells were washed once and fed with fresh medium; 48 h after transduction, cells were collected and analyzed by flow cytometer (Attune, Fisher Scientific, Hampton, NH, USA) or by measuring gaussia luciferase activity using a Microplate reader (FilterMax F5, Molecular Devices, San Jose, CA, USA). For primary CD4^+^ T cells, cells were first transduced with shRNA control or pooled shRNA IFITM1, 2, 3 stocks by spinoculation. Forty-eight hours after transduction, cells were treated with or without 500 IU/mL IFNα2b for 12 h. Infectious NL4.3 or NL4.3 Env- pseudotypes expressing intron gaussia luciferase reporter (NL4.3 Env/HIV-inGLuc) were then applied and cell media were changed with fresh RPMI1640 (with 20 IU/mL rIL-2). Forty-eight hours after infection/transduction, cells were collected for flow cytometric analysis and virus supernatants were collected for determination of HIV-1 production and infectivity.

### 2.5. Flow Cytometry

CD4^+^ T cells were washed twice with cold PBS plus 2% FBS and incubated on ice with pooled anti-IFITM1, IFITM2 and IFITM3 primary antibodies (1:100 dilution in PBS/FBS buffer) for 1 h. After three washes with 1 × PBS plus 2% FBS, cells were incubated with FITC-conjugated secondary anti-Rabbit IgG for 45 min. Cells were washed twice and fixed with 3.7% formaldehyde for 10 min at room temperature and analyzed by flow cytometry (Attune NxT, Fisher Scientific, Hampton, NH, USA. Results were analyzed using the Flowjo software.

### 2.6. Immunoblotting

Western blotting was performed as previously described [6]. Briefly, cells were lysed in pre-chilled RIPA buffer (1% NP-40, 50 mM Tris-HCl, 150 mM NaCl, 0.1% SDS and protease inhibitor mixture) for 20 min and protein samples were subjected to 10% SDS-PAGE. After proteins were transferred to PVDF membrane, primary antibodies of interest were applied and protein signals were detected using an Amersham Imager 600, GE Health Care.

### 2.7. Statistical Analysis

All statistical analyses were carried out in GraphPad Prism6 (La Jolla, CA, USA), with student t-tests or one-way analysis of variance (ANOVA) used unless otherwise noted. Typical data from at least 3 to 5 independent experiments were used for the analysis.

## 3. Results

### 3.1. Ectopic Expression of IFITMs in U87 Cells Inhibits CXCR4 and CCR5 HIV-1 Entry with Equivalent Efficiency

**Figure 1:**
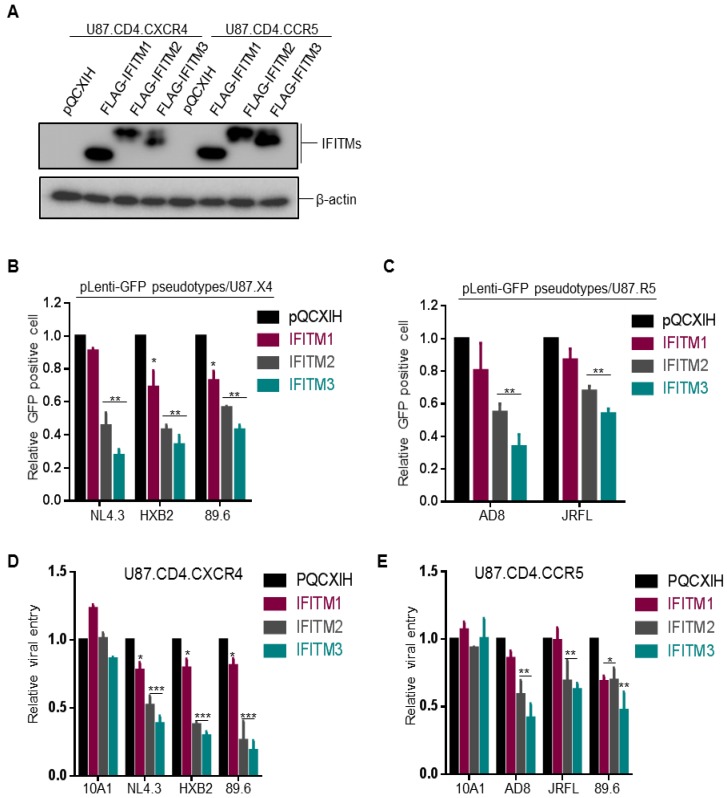
Ectopic expression of IFITM proteins inhibits entry of HIV-1 CXCR4 (X4) and CCR5 (R5) strains at equivalent efficiency independent of co-receptor usage. (**A**) FLAG-tagged IFITM1, 2, 3 expression levels in U87.CD4.CXCR4 and U87.CD4.CCR5 stable cell lines were examined by immunoblotting using an anti-FLAG antibody. β-actin served as loading control. (**B**,**C**) One-round pLenti-GFP HIV-1 infection assay was performed by infecting indicated cells with pLenti-GFP pseudotypes bearing Env of interest and percentages of GFP^+^ cells were quantified by flow cytometry. Relative entry efficiency was calculated by comparing the percentages of HIV-1 GFP^+^ cells expressing IFITMs with that of pQCXIH vector controls. Note that CXCR4 tropic viruses NL4.3 and HXB2 and dual tropic virus 89.6 were examined in U87.CD4.CXCR4 cells (**B**); CCR5 tropic viruses AD8 and JRFL and dual tropic 89.6 were examined in U87.CD4.CCR5 cells (**C**). (**D**,**E**) Single-round infection of HIV-1 NL4.3, AD8 and 89.6 and MLV 10A1 was measured by using HIV-inGLuc reporter pseudotypes in U87.CD4.CXCR4 (**D**) or U87.CD4.CCR5 cells (**E**). See detailed methods in the text. All data are presented as mean ± SD of at least three independent experiments; * *p* < 0.05, ** *p* < 0.01, *** *p* < 0.001.

Neil and colleagues utilized U87.CD4.CXCR4 and U87.CD4.CCR5 cells overexpressing IFITM1, 2 and 3 to investigate the possible differential effects of IFITMs on inhibiting CXCR4 and CCR5 HIV-1 entry and reported that CCR5 HIV-1 was more sensitive to inhibition by IFITM1 while CXCR4 HIV-1 was more sensitive to IFITM2 and IFITM3 [8]. To validate these results, we took the same approach and generated U87.CD4.CXCR4 and U87.CD4.CCR5 cell lines that stably expressed a comparable level of FLAG-tagged IFITM1, IFITM2 and IFITM3 (Figure 1A) and examined the entry of CXCR4-tropic virus strains NL4.3 and HXB2, CCR5-tropic strains AD8 and JRFL, as well as dual tropic strain 89.6.

We first produced GFP-expressing HIV-1 pseudovirions bearing the individual Env of HIV-1 by co-transfecting HEK293T cells with pLenti-Puro-GFP, Gag-Pol Δ8.2 and HIV-1 Env of interest and analyzed the pseudoviral infection efficiency in U87-derived cell lines by flow cytometry. We observed that HIV-1 GFP-pseudotypes bearing the Env of NL4.3, HXB2, 89.6, AD8 or JRFL showed a similar trend of inhibition by three IFITM orthologs, that is, IFITM3 > IFITM2 > IFITM1, regardless of their CXCR4 or CCR5 tropisms (Figure 1B). The extent of inhibition was approximately 2~3 fold for IFITM2 and IFITM3 and <30% for IFITM1, respectively, which were comparable to the original report by Neil and colleagues [8]. We noticed that, while the CCR5-using JRFL HIV-1 was relatively less sensitive to inhibition by IFITM2 and IFITM3 compared with the CXCR4-using viruses HXB2 and NL4.3, another CCR5-using HIV-1 primary isolate, AD8, was as sensitive as HXB2 and NL4.3 to inhibition by IFITM2 and IFITM3. In no case, however, have we found that IFITM1 exhibited a stronger inhibition of any viruses, including CCR5 viruses, than IFITM2 and IFITM3 did (Figure 1B,C). Note that the efficiency of entry by the dual tropic 89.6 in U87.CD4.CCR5 cells was extremely low, as measured by GFP-positive cell population (data not shown); therefore, the result was not informative thus excluded from Figure 1C.

The modest inhibition of IFITMs on entry of different HIV-1 strains shown above (2~3-fold) made us to speculate that perhaps the window of flow cytometry-based entry assay might be too narrow to distinguish the possible difference between CCR5 and CXCR4 viruses inhibited by different IFITMs. To address this potential problem, we took advantage of the recently developed intron-gaussia luciferase-based system for HIV (HIV-inGLuc) [13,14] and re-examined the functional effects of IFITM proteins on CXCR4, CCR5 and dual tropic HIV-1 strains in U87 ells. The HIV-inGLuc system is highly sensitive and quantitative compared to the classical colorimetric assays, including fluorescence imaging and flow cytometry; and because the expression of inGLuc solely relies on infection of new target cells, attributed to an insertion of antisense orientation of Gluc cassette in reporter vector [14], this assay measures a single-round HIV-1 infection.

Similar to GFP-expressing lentiviral pseudotypes described above, we produced inGLuc-reporter viruses by co-transfection of HEK293T cells with an HIV-inGLuc (Env-deficient) vector, a packaging vector Δ8.2 encoding HIV-1 Gag-Pol and all accessory/regulatory genes, along with the Env construct of interest, including murine leukemia virus (MLV) 10A1 Env constructs; 10 A1 was known to be refractory to IFITM restriction; hence, it served as a negative control [10]. As shown in Figure 1D,E, IFITM2 and IFITM3 were more potent than IFITM1 to suppress infection of CXCR4-tropic viruses NL4.3 and HXB2 in U87.CD4.CXCR4 cells, infection of CCR5-tropic viruses AD8 and JRFL in U87.CD4.CCR5 cells, as well as infection of the dual tropic HIV-1 89.6 in both U87.CD4.CXCR4 and U87.CD4.CCR5 cells, regardless their co-receptor usages. As would be expected, IFITM2 and IFITM3 showed no significant effect on 10A1 MLV infection, yet IFITM1 slightly enhanced 10A1 infection (Figure 1D,E). Similar to GFP-expressing HIV-1 pseudotypes shown above, we found that JRFL was relatively resistant to IFITMs, that is, almost no inhibition by IFITM1 and a modest inhibition by IFITM2 and IFITM3 (Figure 1E). 

We noticed that infection by the dual tropic HIV-1 89.6 in U87.CD4.CCR5 cells was almost equivalently inhibited by IFITM1 and IFITM2; however, IFITM3 still exhibited the strongest inhibition among all three IFITMs (Figure 1E). Additionally, the infection efficiency of the dual tropic 89.6 HIV-1 in both U87.CD4.CXCR4 and U87.CD4.CCR5 parental cells was much lower than other strains of HIV-1 and this was particularly the case for infection of HIV-1 89.6 in U87.CD4.CCR5 cells, which was approximately 2~3-fold above the control background. Overall, our results did not support the notion that IFITM proteins restrict HIV-1 entry dependent of co-receptors CCR5 and CXCR4 usage.

### 3.2. Entry of T/F Viruses in U87 Cells Is More Sensitive to Inhibition by IFITM2 and IFITM3 than by IFITM1

**Figure 2:**
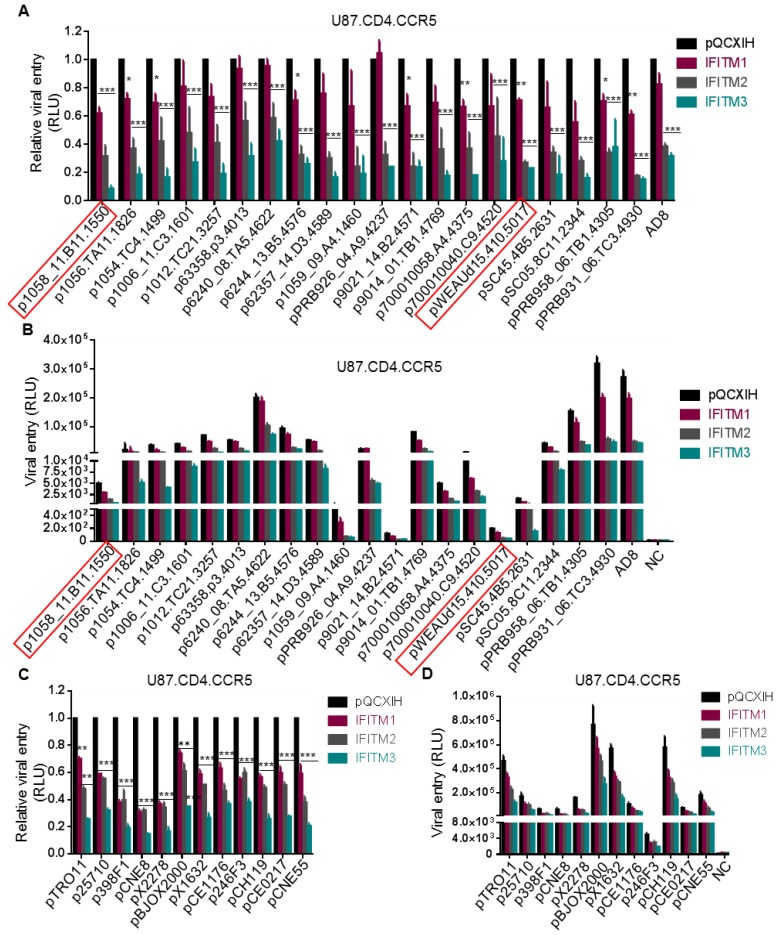
Entry of T/F viruses is more sensitive to inhibition by IFITM2 and 3 than by IFITM1. Twenty subtype B HIV-1 Envs from American countries (**A**,**B**) and twelve global (**C**,**D**) T/F HIV-1 Envs were used to pseudotype the HIV-inGLuc vector and their entry into U87.CD4.CCR5 cells was determined by measuring the gaussia luciferase activity. The relative (**A**,**C**) and absolute (**B**,**D**) comparisons of viral infection, indicative of entry, were plotted, respectively. Red squares indicate two dual tropic T/F viruses. Relative entry was calculated by comparing the gaussia luciferase activities of IFITM-expressing cells with those of pQCXIH vector control; data are mean ± SD of at least three independent experiments; * *p* < 0.05, ** *p* < 0.01, *** *p* < 0.001.

We next examined a panel of HIV-1 subtype B transmitted founder (T/F) Env constructs (NIH AIDS Reagent Program) using the highly sensitive inGLuc system described above and the results were summarized in Figure 2A,B. Overall, IFITM3 showed the strongest inhibition on entry of all T/F Env-mediated HIV-1 into U87.CD4/CCR5 cells, that is, approximately by 2~8-fold compared to vector controls (Figure 2A). IFITM1 had the least effect, about 0–40% reduction relative to the mock control (Figure 2A). The inhibitory effect of IFITM2 was between IFITM1 and IFITM3, although in several cases IFITM2 and IFITM3 exhibited an equivalent inhibitory inhibition (Figure 2A).

The absolute infection efficiency of these T/F viruses in U87.CD4.CCR5 cells, as measured by the gaussia luciferase activity, was plotted in Figure 2B. We noticed that three T/F HIV-1 strains, that is, p6240_08.TA5.4622, pBRB958_06.TB1.4305 and pBRB931_06.TC3.4930 infected U87.CD4.CCR5 cells with an efficiency comparable to or even higher than that of AD8 (Figure 2B). Eleven of these T/F viruses had an intermediate infection efficiency, with luciferase activity ~500 fold above the mock control (Figure 2B). The rest six T/F viruses showed a very low efficiency of infection, especially p1059_09.A4.1460, p9012_14.B2.4571, p1058_11.B11.1550 and pWEAUd15.410.5017; the latter two were dual tropic Env clones and exhibited a gaussia luciferase activity approximately 5~20-fold above the background.

We further tested HIV-1 entry mediated by a panel of 12 global T/F Envs (NIH AIDS Reagent Program) that cover subtypes A, B, C, G, AE, BC and AC in U87.CD4/CCR5 cells expressing different IFITM orthologs. As shown in Figure 2C, IFITM3 displayed the strongest inhibition on all of these T/Fs, that is, up to 5-fold reduction; IFITM2 showed an intermediate level of inhibition (about 2~3-fold reduction); and IFITM1 exhibited the least effect (20–50%). Similar to the results of the subtype B Env panel, the absolute rates of infection for these global Env pseudotypes also varied, with pBJOX2000, pX1632 and pCH119 T/F strains highly efficient yet other T/F viruses, in particular p246F3, much less efficient (Figure 2D).

Taken all the data from subtype B and global Env panels together, we concluded that HIV-1 T/F strains are sensitive to restriction by IFITMs, with efficiency of inhibition ranged from high to low as IFITM3 > IFITM2 > IFITM1, in U87.CD4.CCR5 cells. These results differed from some of those shown by Neil and colleagues [8].

### 3.3. Expression of IFITM Proteins in HuT78, SupT1 and THP-1 Cells Equivalently Inhibits CXCR4 and CCR5 HIV-1 Entry

**Figure 3:**
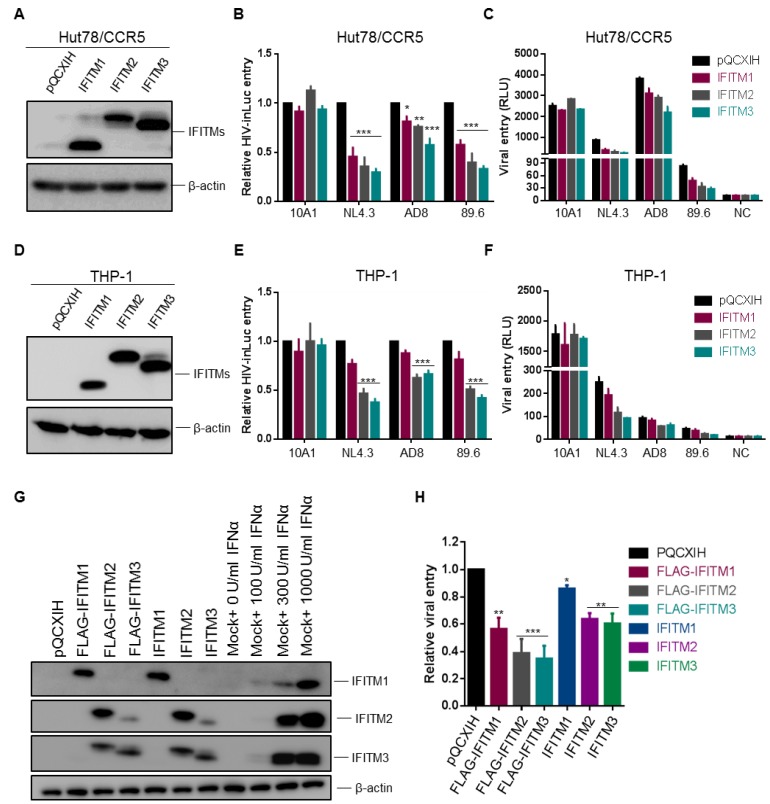
Ectopic expression of IFITM proteins in CD4+ HuT78 and THP-1 cells equivalently inhibits entry of HIV-1 CXCR4 and CCR5 isolates. Experiments were performed the same as described for Figure 1 and Figure 2, except that HuT78 and THP-1 cells expressing individual IFITMs were used for infection. (**A**) IFITM1, 2, 3 expression levels in HuT78/CCR5 cells were examined by immunoblotting using an anti-FLAG antibody. (**B**,**C**) Infection of HIV-inGLuc bearing the Env of MLV 10A1, HIV-1 NL4.3, AD8 or 89.6, indicative of entry, in HuT78/CCR5 cells was shown. Relative entry efficiency was shown in (**B**) and absolute luciferase activities were plotted in (**C**). (**D**) IFITM1, 2, 3 expression levels in THP-1/CCR5 cells were examined by immunoblotting using an anti-FLAG antibody. (**E**,**F**) Infection of HIV-inGLuc bearing the Env of MLV 10A1, HIV-1 NL4.3, AD8 or 89.6, indicative of entry, in THP-1/CCR5 cells was presented. Relative entry efficiency was shown in (**E**) and absolute luciferase activities were plotted in (**F**). (**G**) Expression of FLAG-tagged and untagged wildtype (WT) IFITMs in SupT1 cells, as well as their comparisons with endogenous IFITMs in SupT1 cells induced by IFNα2b treatment (0, 100, 300 and 1000 U/mL). β-actin served as loading control. (**H**) Effect of FLAG-tagged and WT IFITMs on HIV-1 NL4.3 inGLuc infection in SupT1 cells determined by gaussia luciferase activities 48 h after infection. Data are mean ± SD from at least three independent experiments; * *p* < 0.05, ** *p* < 0.01, *** *p* < 0.001.

The U87 cell lines used by Neil and colleagues [8], as well as in our experiments shown above, are neuroblastoma-derived cells, which are not the natural targets of HIV-1 infection. We next sought to determine if the above results obtained from U87 cells could be corroborated in CD4^+^ T cells and monocytes. To achieve infection of both CXCR4 and CCR5 HIV-1 strains, we utilized a HuT78 cell line, which expresses an endogenous level of CD4 and CXCR4 [15] but was transiently transduced to express CCR5 and different IFITMs. After being confirmed to express an equal level of IFITM proteins in these cells as shown in Figure 3A, these cells were infected with HIV-inGLuc reporter viruses bearing Env proteins of NL4.3, AD8 or 89.6. We observed that, IFITM3 and to a lesser extent IFITM2, was more potent than IFITM1 to inhibit entry mediated by NL4.3, AD8 and 89.6 Env (Figure 3B), regardless of their co-receptor preferences. As would be expected, IFITMs had no effect on entry mediated by 10A1 MLV Env (Figure 3B,C). Of note, AD8 Env-mediated entry in HuT78/CCR5 cells was relatively resistant to all three IFITM orthologs compared with NL4.3 and 89.6 (Figure 3B), which was consistent with previously published results [7]. The efficiencies of NL4.3, AD8 and 89.6 infection, as measured by their original luciferase activities in parental Hut-78/CCR5 cells, were plotted in Figure 3C, showing that AD8 had the highest rate of infection, ~4~fold and ~50-fold above HIV-1 NL4.3 and 89.6, respectively.

We next examined entry of NL4.3, AD8 and 89.6 pseudotypes in monocyte-derived CD4^+^ THP-1 cells stably expressing IFITMs. The expression levels of IFITM1, IFITM2 and IFITM3 in these THP-1 cells were comparable, as confirmed by western blotting (Figure 3D). Interestingly, we found that IFITM2 and IFITM3 proteins were almost equally efficient, as compared with IFITM1, to inhibit entry of all three HIV-1 pseudotypes, despite that AD8 was relatively resistant to all three IFITMs (Figure 3E). The absolute infection efficiency of these pseudotyped viruses in these THP-1 cells is shown in Figure 3F, again with NL4.3 being the highest, compared with AD8 and 89.6, which were 3~6-fold above the background control. Accumulatively, these data showed that IFITM2 and IFITM3 are strong inhibitors of HIV-1 entry in CD4^+^ HIV-relevant T lymphocytes and monocytes, similar to what was observed in U87 cells.

To determine if FLAG-tagged IFITM constructs used in above experiments had the same antiviral activities as their untagged wildtypes (WTs), we generated SupT1 cells stably expressing FLAG-IFITMs and WT IFITMs, respectively. We first compared the expression levels of these IFITMs in SupT1 cells, in parallel with that of parental SupT1 cells treated with different doses of IFNα2b. We observed that the levels of FLAG-tagged IFITMs were comparable to those of untagged IFITMs, the levels of both corresponded to approximately 300 U (for IFITM2/3) or 1000 U (for IFITM1) IFNα2b-induced endogenous IFITMs in SupT1 cells (Figure 3G). We next examined the effect of these FLAG-tagged IFITMs and WT IFITMs on infection of SupT1 cells by using HIV-1 NL4.3 inGLuc pseudotypes and observed that FLAG-tagged IFITMs were generally 25–30% more potent to inhibit HIV-1 infection compared with the corresponding WT IFITMs (Figure 3H). However, the trend of inhibition in term of potency by WT IFITMs was the same as that of FLAG-IFITMs, that is, IFITM3 > IFITM2 > IFITM1.

### 3.4. Endogenous IFITM Proteins in CD4^+^ T Cells Play Critical Role in IFN-Mediated Restriction of HIV-1 Replication but Contribute Less at the Entry Level

**Figure 4:**
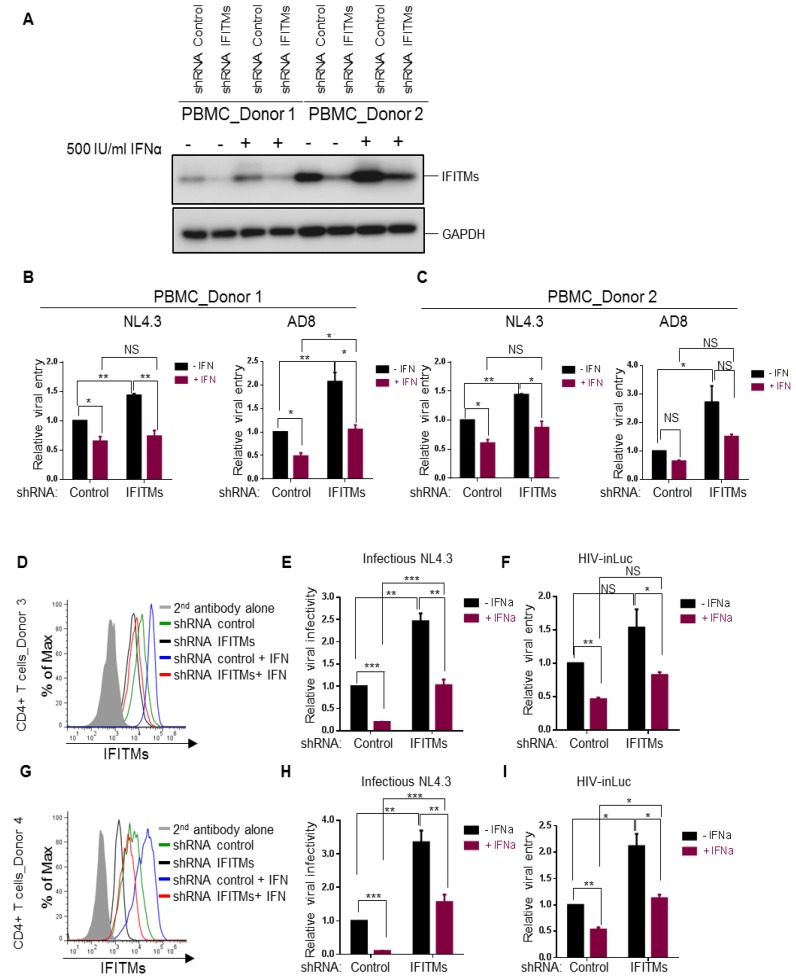
Effects of type I IFN and endogenous IFITM proteins on HIV-1 entry and replication in human PBMCs and CD4^+^ cells. (**A**–**C**) PBMCs from two healthy human donors were activated by 20 IU/mL IL-2 and 5 µg/mL PHAP for 3 days and were transduced with lentiviral shRNA control or pooled shRNA IFITMs for 24 h. Cells were then treated with 500 IU/mL IFNα2b for 24 h or left untreated and were infected with HIV-inGluc bearing Env of NL4.3 or AD8 for 24 h before being collected for immunoblotting (**A**), or for measuring the luciferase activities (**B**,**C**). Relative entry was calculated by setting the luciferase activities of cells transduced by shRNA control pseudotypes and untreated with IFNα2b to 1.0. Different comparisons were performed as indicated, with *p* values shown. (**D** through **I**) CD4^+^ T cells were isolated from PBMCs and transduced with lentiviral shRNA control or pooled shRNAs against IFITM1, 2 and 3. Cells were treated with 500 IU/mL IFNα for 24 h or were left untreated. The expression levels of IFITMs were determined by flow cytometry shown in (**D**,**G**) for two different donors. For replication assay, cells were infected with infectious NL4.3 (**E**,**H**) for 48 h and harvested virions were used to infect HeLa-TZM cells, with firefly luciferase activities being measured 24–48 h after infection. For one-round entry assay, cells were infected with HIV-inGLuc reporter viruses (**F**,**I**) and gaussia luciferase activities were directly measured from the harvested supernatants. Relative infectivity and relative entry were determined by setting the values of shRNA control and IFNα-untreated cells to 1.0. Data were mean ± SD of three experiments, each performed in duplicates; * *p* < 0.05, ** *p* < 0.01, *** *p* < 0.001.

We next interrogated the roles of endogenous IFITM proteins in modulating HIV-1 infection in primary human CD4^+^ PBMCs and purified T lymphocytes. Because short-hairpin RNAs against IFITMs (shRNA IFITMs) exhibit cross-knockdown activities against different IFITM orthologs [6], making the evaluation of the contributions of individual IFITMs to HIV-1 entry challenging, we decided to knock down all three IFITMs altogether by using a mixed pool of shRNAs and compared the overall function of IFITMs on infection of CXCR4 and CCR5 HIV-1 strains, i.e., NL4.3 and AD8, respectively. Human PBMCs of two healthy donors were transiently transduced with lentiviral pseudovirions expressing a mixed pool of human IFITM shRNAs (IFITM1, 2 and 3) and the IFITM knockdown efficiency, in the presence or absence of IFNα2b, was assessed and confirmed by western blotting shown in Figure 4A. Single-round infection assays using inGLuc pseudotypes showed that in the absence of IFNα2b, knockdown of IFITMs in donor 1 and donor 2 (Figure 4B,C) modestly increased HIV-1 NL4.3 entry (~50%), yet more potently elevated entry of AD8 (~2–3-fold). Notably, treatment of PBMCs with IFNα2b decreased NL4.3 and AD8 entry by roughly 2-fold in the shRNA control, yet had very modest effect, approximately 30–50% increase, on PBMCs treated with shRNA IFITMs (Figure 4B,C). These results suggested that IFITMs do not significantly contribute to the IFN-mediated inhibition of HIV-1 entry and implied that other ISGs as yet to be discovered in target PBMCs are likely involved in restricting HIV-1 entry. The ~2-fold increase in HIV-1 entry as a result of IFITM knockdown was actually consistent with the ~2–3-fold inhibition by IFITM overexpression in most cell lines, including U87, HuT78, SupT1 and THP-1 cells shown above. 

The modest effect of type I IFN, especially IFITMs, on HIV-1 entry observed above prompted us to directly compare the effects of type I IFN on one-round HIV infection (based on inGLuc) and multiple-around HIV-1 replication (based on viral infectivity in HeLa-TZM cells). We purified human CD4^+^ T cells from PBMCs of two additional donors, donor 3 and donor 4, and utilized the same set of shRNAs to knock down IFITMs in purified CD4^+^ cells; cells were subsequently treated with IFNα2b for 24 h before being infected with HIV-inGLuc or infectious NL4.3. The knockdown efficiency of IFITMs was evaluated and confirmed by flow cytometry using mixed anti-IFITM antibodies shown in Figure 4D,G. We observed that IFITM proteins were indeed induced by type I IFN and that treatment of cells with IFITM shRNAs efficiently knocked down the IFITM protein expression (Figure 4D,G). Type I IFN greatly suppressed the replication of HIV-1 NL4.3 in shRNA control cells, that is, by 5- and 10-fold in donor 3 and donor 4, respectively, and knockdown of IFITMs attenuated the IFN-mediated restriction of HIV-1 replication to 2~2.5-fold (Figure 4E,H), implicating that IFITMs indeed play a critical role in IFN-mediated restriction of HIV-1 replication. In one-round HIV-1 inGLuc infection assays, which was performed side-by-side, we observed a ~2-fold reduction of HIV-1 infection in IFNα2b-treated CD4^+^ T cells of both donors and that knockdown of IFITMs did not significantly rescue the IFN-mediated inhibition on HIV-1 infection (approximately 50%) (Figure 4F,I), suggesting that endogenous IFITMs in target CD^+^ T cells do not significantly contribute to IFN-mediated inhibition of HIV-1 entry. Taken together, these data, combined with previous reports from our group and others showing that IFITMs potently impaired HIV-1 infectivity in viral producer cells [4,5,6], reinforce the notion that IFITMs do not drastically block HIV-1 entry in target CD4^+^ cells, which explained, in part, why HIV-1 was originally not found to be sensitive to IFITM restriction by Brass et al. [16].

## 4. Discussion

IFITMs are one of the few ISGs that have been characterized to block viral entry [1,2,17]. Although the detailed antiviral mechanisms remain elusive, it is generally believed that expression of IFITMs changes the local lipid property and/or membrane mechanics, thus inhibiting virus fusion with the host cell membrane [10]. However, recent studies from HIV and other viruses have suggested additional mechanisms of action for IFITMs, that is, IFITMs can act in viral producer cells and/or viral particles to impair viral infectivity [4,5,6,17,18]. Even more interestingly, IFITMs have been shown to enhance human CMV assembly and human coronavirus OC43 entry, although the exact mechanisms of which are currently unknown [19,20].

Earlier investigations into the effect of IFITMs on HIV-1 have mainly focused on the effects of IFITM proteins on entry in target cells and revealed generally no or moderate inhibition of HIV-1 entry [16,21]. However, long-term HIV-1 replication assay revealed that IFITM proteins indeed potently suppress HIV-1 spread in SupT1 and other cells, as well as block cell-to-cell transmission [3,5,6,22,23]. The discrepancy was partially resolved by several recent studies, including ours, showing that IFITM proteins mainly act in viral producer cells and/or in IFITM-bearing virions by diminishing HIV-1 infectivity [4,5,6]. In a recent study, our collaborator Chen Liang’s group has found that some CCR5 viruses, including AD8, YU-2 and certain T/F viruses, are relatively resistant to IFITM-mediated inhibition of HIV-1 infectivity compared with some CXCR4 viruses, such as NL4.3 and dual tropic 89.6 [7]. Interestingly, a recent study by Neil and colleagues reported that the effect of IFITMs on HIV-1 entry in target cells is co-receptor dependent, showing that IFITM1 preferentially inhibited entry of CCR5-using HIV-1, including T/F viruses, whereas IFITM2 and 3 were more potent to block entry of CXCR4-using HIV-1 isolates [8], which differed from some of the previous reports [4,6,7,24]. To clarify the discrepancies for the effect of IFITMs on HIV-1 entry, in this work we generated a set of U87.CD4 cell lines expressing either CCR5 or CXCR4, along with different IFITMs similar to those reported by Neil and colleagues and applied GFP as well as intron gaussia luciferase reporter viruses to examine the effects of different IFITMs on the entry of some representative HIV-1 strains and primary isolates. In addition to U87 cells, we also tested the effect of IFITMs on HIV-1 infection of more physiologically relevant human CD4^+^ T cells and monocytes. We observed that, despite the comparable IFITM1, 2 and 3 expression in different cell line systems, the patterns of IFITM inhibition on HIV-1 entry—as ranked by their potency of inhibition—remained the same, that is, IFITM3 > IFITM2 > IFITM1, irrespective of the virus strains tested and the cell lines used.

It has been generally agreed that IFITM1 is expressed predominantly on the plasma membrane and thus more potently inhibit entry of viruses that fuse on the cell surface; in contrast, IFITM2 and IFITM3 are predominantly localized in the endosomal membranes, thus more potently inhibiting viruses that enter cells through endocytosis. In this respect, our results may favor a model that HIV-1 fusion predominantly occurs in endosome, as having been shown by Melikyan’s group and others, regardless of co-receptor usages [25,26]. However, we must stress that this mechanism of HIV-1 entry currently remains controversial [27,28,29,30]. Furthermore, the subcellular localization of IFITM2 and 3 in endosomes may not necessarily be associated with their inhibition of HIV-1 entry [31,32]. It is possible that IFITM1 is intrinsically weaker than IFITM2 and IFITM3 to suppress entry of HIV-1 and other viruses [16,33,34], regardless of the viral entry pathways. It has been frequently demonstrated that overexpression of IFITM1 can result in its abundant expression in endolysosomes, in addition to the plasma membrane; however, its effect on entry of many viruses tested still remains poor, supporting that the intrinsic less potent effect of IFITM1 on entry of HIV-1 and other viruses [2,35] may be due to its extended C-terminal amino acid sequence [6,36,37].

One of the most exciting yet highly controversial findings over the past few years is that T/F viruses are more resistant to the host innate immunity, including restriction by IFITMs [38,39]. T/F virus is a single variant selected from a diverse pool of HIV-1 quasispecies during the establishment of early infection and is responsible for establishment of primary infection, bottleneck transmission and virus spread [40,41]. Indeed, we and others have reported that some of HIV-1 T/F strains are relatively resistant to IFITM3-mediated inhibition of viral infectivity [7,8]; however, the detailed effect of IFITMs in target cells on entry of T/F viruses is less clear. In this work, we analyzed the effects of IFITMs on entry of 20 subtype B and 12 global clades T/F viruses (all CCR5 tropic except two dual tropic viruses) by using the highly sensitive HIV-inGLuc system and we observed that despite their diverse entry efficiency, IFITM1 was consistently less potent in restricting entry of all T/F isolates compared with IFITM2 and IFITM3. These results therefore argue against the finding by Foster et al. that IFITM1 preferentially blocks entry of CCR5 viruses [8], including T/Fs. Our results may also suggest that T/F viruses utilize the same entry pathway as that of chronic primary isolates and lab-adapted strains. The fact that some T/F viruses were even more potently inhibited by IFITMs, especially IFITM2 and IFITM3, compared with those of the lab-adapted NL4.3 and HXB2 viruses, argues that IFITM1 may be inherently less potent to block HIV-1 entry. However, we cannot completely rule out the possibility that some of the effect we observed could be cell type-dependent. While it remains to be established whether or not T/F viruses are truly refractory to inhibition by type I IFN, including IFN-induced IFITM proteins, one recent study reported that subtype C T/F viruses failed to show higher replicative capacity and resistance to IFN-mediated inhibition [39]. This finding, along with some others [42,43,44], raise the possibility that T/F viruses may not be universally refractory to type I IFN as having been previously thought [38]. Using the perfectly matched T/F viruses and chronic viruses and testing them in different and more physiologically relevant cell systems are necessary to address this important issue.

It is still of interest but also puzzling as to why IFITM proteins expressed in our system behaved differently from those of Foster et al., even in the same type of target U87 cells [8]. We speculated that this could be due to the expression levels of IFITM proteins in our system versus those in Neil and colleague’s study [8]. In addition, the N-terminal FLAG-tag in our IFITMs could contribute to some of the differences observed. Indeed, we have observed that FLAG-tagged IFITMs are approximately 25–30% more potent than their WTs to inhibit HIV-1 infection; however, the order of potency in inhibition for the untagged WT IFITMs and FLAG-IFITMs remains the same, that is, IFITM3 > IFITM2 > IFITM1. Another possibility, which also needs to be explored, is whether or not some HIV-1 accessory proteins present in pseudotyped viruses could influence the effect of different IFITMs on HIV-1 infection.

In this work, we also examined the role of endogenous IFITMs in HIV-1 entry and compared with their effect on HIV-1 replication in parallel. We found that knockdown of IFITMs had modest effect on rescuing IFN-mediated inhibition of HIV-1 entry, in contrast to its stronger effect on viral replication. In addition, we showed that the type I IFN itself was not highly effective to block HIV-1 entry, as compared its suppression of HIV-1 replication—based on results from both PBMCs and purified CD4^+^ T cells. Overall, the results presented here, along with our previous study that compared the functional role of IFITMs in viral producer cells and target cells in parallel [6], led us to the conclusion that the predominant effect of IFITMs on HIV-1 is in viral producer cells that diminishes the viral infectivity but not in target cells to block viral entry. This conclusion is also supported by several recent studies [4,5,6] and explains, at least in part, why HIV-1 was originally not found to be restricted by IFITMs [16,21].
